# News and Notes

**Published:** 1907-02

**Authors:** 


### NEWS AND NOTES.

After twenty years' existence the Brooklyn Medical Journal has discontinued publication with the December issue. Its place will be to some extent supplied by the new Long Island Medical Journal, under the editorship of Dr. Paul Pilcher, representing the Associated Physicians of Long Island.

A reception in honor of Dr. and Mrs. William Osler was given at the residence of Dr. and Mrs. Harvey Cushing, December 10th.

A mass-meeting of the alumni of the University of Maryland was held in Baltimore, Md., Jan. 22nd, at 8 P.M., to arrange for the Centennial Celebration of the University of Maryland to be held May 30th to June 2nd, 1907.

The Norwegian bark Medbor, which foundered near Sapelo two weeks previously, landed in Savannah, January 20th, having aboard a number of the crew sick with beri beri. The Medbor sailed from Port Natal several months ago and owing to ship-wreck and quarantine has been greatly delayed. Two of the crew died of beri beri.

A public health conference was held in the Hudson Theater in New York City, with the result that a national society was formed for the purpose of combatting the adulteration of foods and drugs, the sale of alcohol and opium in the guise of "patent" medicines, the practice of the quack and the charlatan, both in and out of the medical profession, the use of the United States mails for fraudulent and indecent advertising, and other dangers of a like kind to the public health and morals. Many delegates from medical, philanthropic, religious, and charitable organizations in all parts of the Union, were present.

---

The University of North Carolina has opened its new chemical building, in which will be located the laboratories of pathology, bacteriology and physiology.

---

Efforts are being made by the medical profession of London to induce the government to have hygiene and temperance taught in the elementary schools by medical men who understand the subjects.

---

A call has been issued for a re-union of the class of 1901 of the University of Maryland, to be held during the Centennial exercises May 30th to June 3rd, 1907, in Baltimore, Md. A muster of the list of graduates of this year shows that only 51 of the 71 men can be located. Further information concerning this meeting may be obtained from Nathan Winslow, University Hospital, Baltimore.

---

Physicians who are interested in the study and legitimate practice of physical (drugless) therapeutic methods, notably electro-therapy, photo-therapy, mechano-therapy, hydro-therapy, suggestion and dietetics, are invited to join the American Physio-Therapeutic Association. Address the Secretary: Dr. Otto Juettner, No. 8 W. Ninth St. Cincinnati, Ohio.

The officers for the ensuing year are:

President: Dr. H. H. Roberts, Lexington, Ky.

Secretary: Dr. Otto Juettner, Cincinnati, Ohio.

Treasurer: Dr. Geo. H. Grant, Richmond, Ind.

Executive Council: Drs. W. F. Klein, Lebanon, Pa.; Jas. Hanks, Brashear, Mo.; J. W. Unger, West Point, Miss.; Chas. L. Northen, Talladega, Ala.; R. W. Gibbes, Columbia. S. C.; S. J. Crumbine, Topeka, Kans.; F. L. Keeler, Perry, Okla.

